# Validity and Concordance of a Linear Position Transducer (Vitruve) for Measuring Movement Velocity during Resistance Training

**DOI:** 10.3390/s24196444

**Published:** 2024-10-05

**Authors:** Jaime González-Galán, José Carlos Herrera-Bermudo, Juan José González-Badillo, David Rodríguez-Rosell

**Affiliations:** 1 Department of Sport and Computer Science, Universidad Pablo de Olavide, 41013 Seville, Spain; jaimegonzalezgalan99@gmail.com (J.G.-G.); josecarloshb99@gmail.com (J.C.H.-B.); 2 Physical Performance & Sports Research Center, Universidad Pablo de Olavide, 41013 Seville, Spain; jjgbadi@gmail.com; 3 Research, Development and Innovation (R&D+i) Area, Investigation in Medicine and Sport Department, Sevilla Football Club, 41005 Seville, Spain

**Keywords:** validity, reliability, velocity-based resistance training, linear position transducer, measurement

## Abstract

This study aimed to analyze the intra-device agreement of a new linear position transducer (Vitruve, VT) and the inter-device agreement with a previously validated linear velocity transducer (T-Force System, TF) in different range of velocities. A group of 50 healthy, physically active men performed a progressive loading test during a bench press (BP) and full-squat (SQ) exercise with a simultaneous recording of two VT and one TF devices. The mean propulsive velocity (MPV) and peak of velocity (PV) were recorded for subsequent analysis. A set of statistics was used to determine the degree of agreement (Intraclass correlation coefficient [ICC], Lin’s concordance correlation coefficient [CCC], mean square deviation [MSD], and variance of the difference between measurements [VMD]) and the error magnitude (standard error of measurement [SEM], smallest detectable change [SDC], and maximum errors [ME]) between devices. The established velocity ranges were as follows: >1.20 m·s^−1^; 1.20–0.95 m·s^−1^; 0.95–0.70 m·s^−1^; 0.70–0.45 m·s^−1^; ≤0.45 m·s^−1^ for BP; and >1.50 m·s^−1^; 1.50–1.25 m·s^−1^; 1.25–1.00 m·s^−1^; 1.00–0.75 m·s^−1^; and ≤0.75 m·s^−1^ for SQ. For the MPV, the VT system showed high intra- and inter-device agreement and moderate error magnitude with pooled data in both exercises. However, the level of agreement decreased (ICC: 0.790–0.996; CCC: 0.663–0.992) and the error increased (ME: 2.8–13.4% 1RM; SEM: 0.035–0.01 m·s^−1^) as the velocity range increased. For the PV, the magnitude of error was very high in both exercises. In conclusion, our results suggest that the VT system should only be used at MPVs below 0.45 m·s^−1^ for BP and 0.75 m·s^−1^ for SQ in order to obtain an accurate and reliable measurement, preferably using the MPV variable instead of the PV. Therefore, it appears that the VT system may not be appropriate for objectively monitoring resistance training and assessing strength performance along the entire spectrum of load-velocity curve.

## 1. Introduction

Monitoring relative load and volume during resistance training (RT) is crucial to determine the magnitude and orientation of training-induced adaptations [1,2,3]. For this reason, it is necessary to use precise, objective, and valid procedures that allow these variables to be quantified during resistance exercises [4]. Velocity-based RT (VBRT) is considered a novel approach founded on monitoring repetition movement velocity for assessing, programming, and dosing the degree of effort during RT [2]. The close relationship observed between the movement velocity and the %1RM allows us to use the lifting velocity of the first repetition of the set as a considerably accurate and valid estimator of the relative load (provided that each repetition is conducted at maximal intended velocity) [2,5,6,7]. In addition, the percentage of velocity loss (percentage decrease in movement velocity between the fastest and the last repetition in the set) is directly related to the percentage of repetitions performed with respect to the maximum number of repetitions that can be completed during a set against a given relative [2,8,9] and its magnitude determine the acute metabolic stress, hormonal response, and mechanical fatigue [10,11,12,13]. Thus, by monitoring the velocity of the first repetition and the velocity loss in each training set, it would be possible to know with considerable precision the degree of actual effort exerted by individuals in each RT session [2,8]. Therefore, it is essential to have accurate, objective, and valid velocity-measuring instruments to guarantee adequate training control.

Currently, there are a large number of commercially available devices used for measuring and recording movement velocity, including linear velocity (LVT) and linear position transducers (LPT), accelerometers, smartphone apps, 3D filming systems, optoelectronic cameras, or smartwatches. The proliferation of measurement instruments has allowed VBRT to be more accessible to coaching, strength and conditioning professionals, scientists, and professional and non-professional athletes. The choice of which device to use may be conditioned by multiple factors, including measured variables, sampling frequency, external power supply required, time needed to obtain the measurement after execution, data visualization, portability, and price. However, the most important criterion to be considered in a measuring device for VBRT should be the validity (i.e., accurately and reliably measure the execution velocity), regardless of the practitioners. This is because small variations in the velocity against different loads involve relevant changes: (1) in the relative load or degree of effort experienced during training, and (2) in the strength performance status of individuals during an assessment.

Several studies have analyzed and compared the reliability and validity of different velocity measuring devices. Although a myriad of wearable sensors has been developed and commercialized for providing velocity feedback during RT, controversy still remains regarding the reliability and validity of these devices [14,15,16,17,18,19]. For this reason, the most common devices used for measuring movement velocity are the LPT (derives velocity from the recorded displacement-time data using the inverse dynamic approach) and LVT (directly provides velocity measurements through the recording of electrical signals that are proportional to the cable’s extension velocity) [3,20]. In general, these instruments have shown high intra-device agreement and reliability and low magnitude of error [14,17,18,21,22]. However, LPT and LVT are considered expensive (>USD 1500) and may not be practical outside of laboratory settings because they need to be connected to a PC. Fortunately, the implementation of wireless technology, Bluetooth and Wi-Fi, and the reduction in material costs has increased their portability, simplicity, and accessibility, although the available data regarding the reliability and validity of this type of devices are scarce [14,17,18,23]. In this line, Vitruve (Vitruve, Madrid, Spain) is a relatively new LPT with a considerably lower price (USD 490) that allows data transmission (100 Hz) via Bluetooth and can be viewed in real time through a specific app available for Android or iOS. To our knowledge, there is no available data regarding its reliability, agreement, and validity.

Examining the validity of a velocity-measuring device requires the application of appropriate statistical tests in order to reach accurate interpretations and conclusions. Most studies used the Pearson’s correlation coefficient to determine the level of agreement between devices [16,19,24]. However, Pearson’s correlation coefficient is not sensitive to changes in the means of the measurements. This lack of sensitivity means that the correlation value could be very high even if the velocity values of one device are steadily increasing or decreasing their values by any magnitude compared to another device (i.e., not provide any information about systematic errors inherent in the measurement). Thus, a high correlation coefficient value does not mean a complete agreement between scores [25]. On the contrary, few studies used a Bland–Altman analysis to know and interpret the magnitude of error and established objective criteria of disagreement [14,17,26]. This analysis allows an interpretation of the change in the percentage of the 1RM (% 1RM) that represents a given magnitude of error [14,17,26]. Based on this information, a decision could be made as to whether the device being analyzed is suitable to allow proper implementation of VBRT.

Considering the above, the purpose of this study was to determine the inherent technical errors (i.e., the agreement between two devices from the same model and brand) of a new LPT (Vitruve) and compare their level of agreement/disagreement against a criterion device throughout the whole load-velocity spectrum during the bench press (BP) and full-squat (SQ) exercises.

## 2. Materials and Methods

### 2.1. Study Design

A quantitative, experimental, and cross-sectional design was used in the present study to compare the agreement between three different devices: two LPT (Vitruve System, VT) and one LVT (T-Force Dynamic Measurement System, TF). Additionally, the concordance between both VT systems (VT1 and VT2) and between each VT and TF (reference criterion) system was analyzed. To address this, a group of 50 participants performed 2 testing sessions over 1 week, with at least 72 h of rest. In each testing session, a progressive loading test in the BP (first testing session) and SQ (second testing session) exercises were conducted. The bar velocity of all repetitions was simultaneously recorded with the 3 devices. During the 2 weeks preceding this study, 4 familiarization sessions were undertaken, which consisted of performing several sets with different progressive loads and low velocity loss (<10–15%), executing each repetition at the maximum intended velocity with the proper technique to be used in the testing sessions. All tests were carried out at the same time of the day (±1 h) with constant temperature and humidity room conditions (~20–22 °C and ~55–65% humidity) for each participant. Participants were required not to engage any previous intense physical activity since the day prior of the evaluation session.

### 2.2. Participants

A group of 50 young healthy men volunteered to participate in this study (age: 25 ± 3.3 years; body mass 80.5 ± 6.8 kg; height 179.7 ± 7.6 cm). Participants were physically active sport science students with at least 3 months of previous experience in resistance training, involving the BP and SQ exercises as part of their regular routine. No physical limitations, health problems, or musculoskeletal injuries that could affect the testing were reported. Also, none of the participants were taking drugs, medications, or dietary supplements known to influence physical performance. This study was conducted according to the Declaration of Helsinki and was approved by the local ethics committee. All participants were informed of the procedures and potential risks of performing the tests, and all gave their written consent, signing the informed consent document before taking part in this study.

### 2.3. Testing Procedures

In the first testing session, before starting the warm-up, anthropometric measurements were taken. Height and body mass were determined using a medical scale (Seca 710, Seca Ltd., Hamburg, Germany). The participants were in an anatomical standing position, barefoot, and wearing only underclothes. Each measurement was performed twice, and the average value was taken to define the characteristics of the participants.

### 2.4. Progressive Loading Tests in the BP and SQ Exercises

The warm-up consisted of 5 min of jogging and 5 min of mobility exercise followed by 2 sets of 5 repetitions in the corresponding exercise with 20 kg at a progressively increasing velocity. The initial load for testing was set at 20 kg for all participants and was gradually increased in 10 kg increments until the attained mean propulsive velocity (MPV) was lower than 0.35 m·s^−1^ in the BP and lower than 0.60 m·s^−1^ in the SQ exercise. Three attempts were executed for light (MPV > 0.95 m·s^−1^ for BP and >1.10 m·s^−1^ for SQ), two for medium (0.95 > MPV > 0.55 m·s^−1^ for BP and 1.10 > MPV > 0.80 m·s^−1^ for SQ), and one for heavy loads (MPV < 0.55 m·s^−1^ for BP and <0.80 m·s^−1^ for SQ). Two trained spotters were present when high loads were lifted to ensure safety. Recovery times ranged from 3 min for low loads and 5 min for high loads. In both exercises, participants were carefully instructed to always perform the eccentric phase at a controlled velocity (0.45–0.65 m·s^−1^) and to perform the concentric phase of each repetition at maximal intended velocity. Strong verbal encouragement was provided during each repetition to motivate participants to give a maximal effort.

In the BP exercise, participants laid supine on a flat bench with the back in contact, their feet resting on the bench and hands placed on the bar slightly wider than shoulder width. The position on the bench was carefully adjusted for each subject to ensure that the vertical projection of the bar was 1–2 cm above the intermammary line. This individual position as well as the bar grip width were measured so that they could be reproduced on every lift. The subjects started with their arms fully extended and perpendicular to the trunk, and from this position, they lowered the bar in a controlled velocity until they left it resting on the chest around ~1.5 s and remained waiting until they heard the ‘go!’ command from a researcher. Participants were not allowed to bounce the bar off their chest or raise the shoulders or trunk off the bench. In the SQ exercise, participants started from an upright position, with the knees and hips fully extended and the stance approximately shoulder-width apart with both feet positioned flat on the floor in parallel or slightly externally rotated. The bar was grasped with a closed pronated grip and placed on the upper part of the trapezius, while keeping a stable upright trunk posture. The stance width and feet position were individually adjusted and carefully replicated on every lift. Each participant descended in a uniform and controlled motion until the posterior thighs and calves contacted each other. Then, participants immediately reversed motion and ascended back to the starting position as fast as possible. Participants were not allowed to raise the barbell off the shoulders and to jump off the ground, although subjects were permitted to raise their heels at the end of the concentric phase, which typically occurred when lifting the lighter loads.

### 2.5. Measurement Equipment and Data Acquisition

Testing was performed on a Smith machine (Multipower fitness line, Peroga, Murcia, Spain). This machine permits only vertical displacement of the bar in order to have a smooth and proper lift. The weight of the bar was 20 kg, and the rest was added by sliding calibrated disks (Eleiko, Sport AB, Halmstad, Sweden). The wires of the devices (TF, VT1 and VT2) were located almost together (two centimeters of separation) on the right distal border of the bar in order to be in homogenous conditions. Two velocity outcome measures were obtained from each device in each repetition, which are the following: MPV [27] and peak of velocity (PV). For the subsequent analysis, the fastest repetition obtained with each absolute load (kg) was taken according to the criterion of the highest MPV [5,27].

The LPT were two Vitruve Systems (Vitruve encoder, Madrid, Spain). These systems have a sampling rate of 100 Hz, and the data collected were transmitted through Bluetooth connection to different iPhones 12–iOS 15.0.2 (Apple Inc., California, CA, USA) using the Vitruve app (version 4.23). The VT system recorded displacement-time-curve data by determining changes in the bar position. The LVT was one T-Force Dynamic Measurement System (Ergotech Consulting, Murcia, Spain). Instantaneous velocity values were directly sampled by the device at a frequency of 1000 Hz through a connection to the computer by means of a 14-bit resolution analog-to-digital data acquisition board and custom software (Version 3.60). The TF system smoothed with a 4th order low-pass Butterworth digital filter with no phase shift and a 10 Hz cut-off frequency.

### 2.6. Statistical Analysis

An exhaustive explanation of the statistical analysis conducted has been provided in previous research [14,17]. The normality of the distribution of the variables was examined with the Kolmogorov–Smirnov test. The intraclass correlation coefficient (ICC) was calculated using one-way random effects and a 95% confidence interval (95% CI). Values between 0.95 and 0.99 were established as acceptable for the equipment assessment [28]. The Lin’s concordance correlation coefficient (CCC) was used to detect the agreement and systematic error between devices, and the percent deviation from perfect concordance was also calculated [29]. The agreement based on CCC was interpreted as perfect (CCC = 1), almost perfect (CCC ≥ 0.99), substantial (0.95 ≤ CCC ≤ 0.99), moderate (0.90 ≤ CCC ≤ 0.95), and low (CCC < 0.90) [28,29]. The mean square deviation (MSD) and variance of the difference between measurements (VMD) were calculated as error indicators. A value closer to 0 means a consistent and proportional systematic error and random error in MSD and better precision in VMD. Percent deviation from zero was calculated for both variables [14,17]. Pearson’s correlation coefficient (r) and a linear regression analysis with a standard error of estimate (SEE) were used to analyze the possible relationship between paired velocity values. The standard error of measurement (SEM) was calculated and expressed in absolute and relative terms through the coefficient of variation (CV = 100 × SEM/mean) [30,31]. A CV of less than 10% was established as the criterion for declaring a variable as appropriate for exercise performance tests [31]. Derived from SEM, the smallest detectable change (SDC) was calculated (SDC = √2 × SEM × 1.96) as a measure of sensitivity. Bland–Altman plots were examined to assess the agreement along the entire spectrum of velocity values at each load [32]. Systematic difference (bias) and its 95% limits of agreement (LoA = bias ± 1.96 SD) were calculated [32]. Maximum errors (ME) at the 95% confidence interval were calculated from the SEE (MESEE) and Bland–Altman bias (MEBIAS) for the different velocity outcomes analyzed. Values were expressed as the corresponding relative load (% 1RM) for each velocity and exercise based on previous studies in the BP [5] and SQ [33] exercises. Levels of disagreement were proposed based on clinical considerations and previous published evidence [5,14,17,33,34]. All data analysis were performed using the SPSS statistical software package version 26.0 (SPSS, Chicago, IL, USA). All statistical variables were calculated for the sets of all data (Pooled) and for different velocity ranges within the load–velocity relationship. For the BP exercise, the established ranges (from highest to lowest velocity) were as follows: >1.20 m·s^−1^; 1.20–0.95 m·s^−1^; 0.95–0.70 m·s^−1^; 0.70–0.45 m·s^−1^; and ≤0.45 m·s^−1^. For the SQ exercise, the established ranges were as follows: >1.50 m·s^−1^; 1.50–1.25 m·s^−1^; 1.25–1.00 m·s^−1^; 1.00–0.75 m·s^−1^; and ≤0.75 m·s^−1^.

## 3. Results

A total of 278 and 411 repetitions were performed in the BP and SQ exercise, respectively. The TF recorded all the repetitions (100%) carried out in both exercises. The VT1 recorded 260 (93.5%) and 407 (99.0%) repetitions for the BP and SQ exercises, respectively, whereas the VT2 recorded 250 (89.9%) and 392 (95.4%) repetitions for the BP and SQ exercises, respectively. For the BP exercise, the number of repetitions analyzed in each range of velocity were the following: >1.20 m·s^−1^ = 48; 1.20–0.95 m·s^−1^ = 38; 0.95–0.70 m·s^−1^ = 74; 0.70–0.45 m·s^−1^ = 57; and ≤0.45 m·s^−1^ = 61. For the SQ exercise, the number of repetitions analyzed in each range of velocity were the following: >1.50 m·s^−1^ = 73; 1.50–1.25 m·s^−1^ = 85; 1.25–1.00 m·s^−1^ = 96; 1.00–0.75 m·s^−1^ = 88; and ≤0.75 m·s^−1^ = 69.

The magnitude of error and between-device agreement analysis for the variables MPV and PV obtained during the BP exercise are displayed in Table 1 and Table 2, respectively. When the data were pooled, both variables showed high agreement between VT1 and VT2 with TF (ICC > 0.990, CCC > 0.981, MSD < 1.29%, VMD < 0.7%), and between VT1 and VT2 (ICC > 0.998, CCC > 0.998, MSD < 0.18%, VMD < 0.09%), with a tendency towards a better degree of agreement for the variable MPV compared to PV. However, the ME_SEE_ and ME_BIAS_ between TF and VT devices was moderate (6.4–8.9% 1RM) using the MPV, while for the variable PV the ME was very high (11.4–23.4% 1RM). The ME between VT devices followed the same trend as that shown between TF and VT. When the data were analyzed according to the established velocity ranges, the degree of agreement of VT1 and VT2 with TF decreased as the velocity ranges increased for both variables, being more abrupt for the PV (ICC: 0.754–0.996, CCC: 0.663–0.991, MSD: 5.31–0.03%, VMD: 0.61–0.02%) compared to the MPV (ICC: 0.790–0.992, CCC: 0.698–0.984, MSD: 0.63–0.02%, VMD: 0.10–0.01%). In accordance, the magnitude of error (ME_SEE_ and ME_BIAS_) decreased as the analyzed velocity range decreased (From 7.8 to 2.9% 1RM for the MPV and from 21.8 to 4.2% 1RM for the PV).

For the SQ exercise, the magnitude of error and between-device agreement analysis for the variables MPV and PV are presented in Table 3 and Table 4, respectively. When the data were pooled, high agreement between VT1 and VT2 with TF, and between VT1 and VT2 was observed for the MPV (ICC > 0.998, CCC > 0.996, MSD < 0.10%, VMD < 0.07%), whereas for the PV, the degree of agreement was moderate to high (ICC > 0.966, CCC > 0.937, MSD < 1.65%, VMD < 0.64%). The ME_SEE_ and ME_BIAS_ between TF and VT devices, and between VT1 and VT2 was moderate (5.4–7.5% 1RM) using the MPV, while for the variable PV, the ME was very high (12.1–26.1% 1RM). Similar to the BP exercise, when the data were analyzed by velocity ranges, the degree of agreement of VT1 and VT2 with TF decreased as the velocity ranges increased for both variables, showing worse values for the PV (ICC: 0.695–0.977, CCC: 0.588–0.972, MSD: 4.66–0.12%, VMD: 1.92–0.06%) compared to MPV (ICC: 0.909–0.996, CCC: 0.836–0.992, MSD: 0.25–0.01%, VMD: 0.24–0.01%). The magnitude of error decreased as the analyzed velocity range decreased, with ME_SEE_ and ME_BIAS_ for MPV of ~10–15% 1RM at velocities greater than 1.25 m·s^−1^, between ~4.5–6% 1RM at velocities of 1.25–0.75 m·s^−1^, and ~3% 1RM for velocities <0.75 m·s^−1^. For the PV, the magnitude of error was unacceptable, with some velocity ranges showing ME_BIAS_ of ~42% 1RM.

## 4. Discussion

To the best of our knowledge, this is the first study assessing the inter-device and intra-device agreement of a new LPT (VT) for measuring the MPV and PV in different velocity ranges in the SQ and BP exercises. The main findings of this study were the following: (1) when the data were pooled, VT systems showed high intra- and inter-device agreement in both exercises, with a higher value for the MPV compared to the PV; (2) the intra- and inter-device magnitude of error was moderate for the MPV, whereas the PV was very high in both exercises; (3) the degree of agreement decreased, and the magnitude of error increased as the analyzed velocity range increased (i.e., the load decreases) for both exercises, obtaining worse values for the PV compared to the MVP. For assessing, programming, and dosing the degree of effort during RT, it is strictly necessary to use objective and valid devices throughout the entire spectrum of the load–velocity relationship (i.e., in all velocity ranges). Thus, our results suggest that the VT system may not be an adequate device to implement VBRT.

The marketing of a large number of devices for measuring movement velocity has made VBRT more accessible to coaches and athletes. However, the validity of some devices remains questionable [4,14,17,18,35,36,37]. The lack of agreement with a reference or criterion device (i.e., gold standard) for determining the validity of a new velocity measuring device has been highlighted [14]. Few studies have analyzed the validity of LPT systems, varying the criterion device [14,17,18,35]. Pérez-Castilla et al. [18] observed a high level of agreement between LPT and LVT devices compared to a 3D system (Trio-OptiTrack, Natural-Point, Inc., Corvallis, OR, USA), used as reference criterion. However, this 3D system has not been shown to be more reliable than LPT or LVT devices at the velocity ranges analyzed in the present study (0.34–0.90 m·s^−1^ in PB), which could lead to erroneous conclusions [17]. Specifically, Martinez-Cava et al. [17] showed that, as the velocity range increases, the reliability of these 3D devices tends to decrease, due to the relatively low sampling frequency of the cameras (100–200 Hz). However, TF devices presented higher accuracy and reliability in different exercises and velocity variables compared to LPT, 3D systems, accelerometers, smartphone apps, or optoelectronic cameras [14,17]. Therefore, LVT devices, specifically the TF system, were proposed as a reference criterion device to identify technical and measurement errors of other devices for velocity bar measurements [14,17,18,35].

When the data were pooled, a high inter-device agreement and moderate magnitude of error were observed in both exercises for the MPV (Table 1, Table 2, Table 3 and Table 4). This observation is in accordance with previous studies comparing the validity and concordance of LPT with an LVT device [14,17]. Therefore, it could be concluded that, although a certain magnitude of error was observed, the LPT could be used to measure the repetition velocity (MPV and PV) as a criterion for monitoring RT. However, when the repetitions were analyzed by velocity ranges, the level of agreement decreased, and the magnitude of error increased as the analyzed velocity ranges increased (Table 1, Table 2, Table 3 and Table 4). These results were also in concordance with the same previous studies [14,17]. This issue implies that the measurement would become increasingly inaccurate and less reliable as the relative load decreases. Specifically, for velocities >0.75 m·s^−1^ and >0.45 m·s^−1^ for the SQ and PB exercises, respectively, the VT device exhibits unacceptable magnitudes of error that may limit its use for monitoring and dosing the VBRT.

A previous study indicated that LPT devices could be used as a valid alternative to LVT for measuring movement velocity [18,38], although other studies question its use for medium and high relative loads (MPV > 1.00 m·s^−1^) [17]. Our results showed ICC inter-device values >0.95 for velocity ranges <0.7 m·s^−1^ and <1.25 m·s^−1^ in the PB and SQ exercises, respectively. However, VT only showed acceptable concordance with TF (CCC values > 0.95) when velocity ranges were <0.45 and <0.75 for the BP and SQ exercises, respectively. Moreover, ME values (ME_SEE_ and ME_BIAS_) for VT were only acceptable (ME < 5%1RM) in the last velocity range analyzed (Table 1, Table 2, Table 3 and Table 4). Discrepancies with previous studies may be due to the differences in the analyzed LPT devices. In this regard, it seems that other LPT devices such as Chronojump [14] and GymAware [39] have resulted in acceptable validity compared to the VT system. Therefore, considering both the degree of agreement and magnitude of error, it seems that the VT system should only be used at velocities <0.45 m·s^−1^ and <0.75 m·s^−1^ for the BP and SQ exercises, respectively, which means relative loads >80% 1RM for the BP [5] and >75% 1RM for the SQ exercise [33]. The lack of validity in the measurement, mainly at moderate-high velocity, prevents knowing with precision the relative load used during resistance exercises and the effect induced by the training program.

A high level of agreement and moderate error magnitude was observed for intra- and inter-device analysis in the MPV for both exercises (Table 1 and Table 3), which is in accordance with previous research [14,17]. However, the level of agreement has been moderate to high, and the error magnitude has been high for the PV, especially in the SQ exercise (Table 2 and Table 4). Our results in the PV variable differ considerably from those observed in previous studies with other LPT [14,17], where high values of agreement and low-moderate values of error were provided [14,17]. It is possible that the PV measurement is dependent on the LPT device used. Thus, the sampling rate of the device could be the basis for the signal accuracy for the PV measurement, as previously observed with other measurement systems (e.g., 3D system) [17]. In our study, while the TF sampling rate was 1000 Hz (i.e., 1000 data per second), the VT sampling rate was only 100 Hz (i.e., 100 data per second). Recording a smaller number of data per second (specifically 10 times less) could significantly influence the following: (1) the maximum velocity value recorded (i.e., the PV) due to a smoothing of the velocity–time curve, and (2) the detection point of the start and end of the concentric phase of the movement, conditioning the duration of this phase and, consequently, the MPV calculated by LPT systems. For these reasons, the MPV has been defended as a reference for the determination and monitoring of relative load during VBRT [27].

On the other hand, the lowest values of degree of agreement observed for the SQ compared to the BP exercise may be related to the higher velocity associated with each %1RM, since our results showed a tendency to exhibit lower agreement and higher error magnitude between TF and VT, and between VT systems, as the velocity increases (Table 1, Table 2, Table 3 and Table 4). Another possible explanation for these results could be related to the imposition of a pause (~1.5 s) between the concentric and eccentric phase in the BP exercise compared to the SQ exercise. This momentary pause between phases allows for more reliable and consistent measures [40]. Therefore, it appears that the VT system could have difficulty in identifying the beginning of the concentric phase in SQ exercise due to their lower sampling frequency and no pause between the eccentric and concentric phases of the movement. Thus, our results could suggest that VT systems measure similarly to TF at low velocities (0.75 m·s^−1^ in SQ and 0.45 m·s^−1^ in PB) and only in the MPV variable. In addition, it is noteworthy that differences were observed between VT systems in the number of recorded repetitions (VT1 lost 22 repetitions and VT2 lost 47 repetitions) and in the level of agreement and error magnitude of each VT compared to TF and between VT devices (Table 1, Table 2, Table 3 and Table 4). Considering these results, it is likely that all VT devices contain a certain magnitude of error and that this error may be different in each case. Consequently, the data obtained with different VT units could not be compared.

In brief, the most important criterion to be considered in a measurement device for implementing VBRT should be validity, which implies an accurate and reliable measurement of bar velocity. This is because changes in the MPV of 0.07 to 0.10 m·s^−1^ imply changes in ~5–10% 1RM in the PB and SQ exercises [5,6,33,40], involving relevant changes in the degree of effort exerted during training or in the assessment of individual performance status. For this reason, it is necessary to have a considerably accurate device to ensure that changes in movement velocity are not due to measurement errors. According to our results, the VT devices showed lower agreement and higher degree of error as the range velocity increases. Specifically, the VT system could be valid for very low velocities [MPV < 0.45 m·s^−1^ (~80%1RM) for BP; and MPV < 0.75 m·s^−1^, (~75%1RM) for SQ]. Therefore, it would not be possible to accurately estimate the actual degree of effort of a large part of the load–velocity relationship. In order to completely assess individual performance or training effect and accurately determine the degree of effort during RT, it is necessary to have confidence in measured velocities against a wide spectrum of loads. Thus, it appears that the VT device should not be recommended for monitoring RT.

## 5. Conclusions

The main conclusions of this study were as follows:

- For the BP exercise, VT1 and VT2 showed a high degree of agreement and concordance with TF (reference criterion) for velocities lower than 0.70 m·s^−1^ for both MPV and PV variables. When bar velocities were greater than 0.70 m·s^−1^, the degree of agreement decreased progressively, mainly for the PV variable.
- For the BP exercise, the magnitude of error (for SEE or BIAS) was moderate (~6–7% 1RM) for the MPV, whereas for the PV variable, the magnitude of error was unacceptable (~15–20% 1RM), mainly for velocities >0.70 m·s^−1^.
- For the SQ exercise, the degree of agreement between VT devices and TF for the MPV variable increased as the velocity range decreased, being acceptable for velocities lower than 1.00 m·s^−1^, whereas for the PV variable, the agreement and concordance were moderate to low, even for the low velocity ranges analyzed.
- For the SQ exercise, the magnitude of error (for SEE or BIAS) was moderate (~6–7% 1RM) for MPV at velocity ranges lower than 1.25 m·s^−1^, whereas at velocities greater than 1.25 m·s^−1^, the magnitude of error was unacceptable (~10–15% 1RM). For the PV variable, the magnitude of error was unacceptable (>10% 1RM) for all velocity ranges analyzed.

## 6. Practical Applications

Considering the results obtained in this study, VT may not be a suitable device to monitor RT using the PV variable in the PB and SQ exercises. However, for the MPV variable, VT showed a moderate to high agreement and a low magnitude of error for low velocity ranges in the BP (<0.45 m·s^−1^) and SQ (<0.75 m·s^−1^) exercises. Therefore, it appears that VT could be a useful tool for the measurement of VBRT during training sessions with moderate to heavy relative loads (>70% 1RM), whereas for light loads (<60% 1RM), the magnitude of error that could be made in estimating the relative load can be considered unacceptable. Considering that, in many cases, athletes and other practitioners could and should use moderate to low relative loads during RT sessions, the data recorded with the VT device should be taken with caution to assess the characteristics of the RT and its effects. In addition, the low degree of intra-device agreement found between VT devices suggests that coaches and strength and conditioning professionals should always use the same device to evaluate the strength performance of their athletes or practitioners in order not to introduce a greater error in the measurement.

## Figures and Tables

**Table 1:** Between-device agreement (reproducibility) for the mean propulsive velocity in the bench press exercise according to the velocity range assessed.

	MAGNITUDE OF ERROR					AGREEMENT			
	SEM (m·s^−1^)	SDC (m·s^−1^)	CV (%)	ME_SEE_ (%1RM)	ME_BIAS_ (%1RM)	ICC (CI 95%)	CCC [DEV (%)]	MSD [DEV (%)]	VMD [DEV (%)]
**POOLED**									
TF-VT1	0.0316	0.0877	4.1	6.4	7.8	0.998 (0.997–0.998)	0.996 [0.41]	0.0010 [0.10]	0.0007 [0.07]
TF-VT2	0.0367	0.1018	4.5	6.4	8.9	0.995 (0.994–0.996)	0.998 [0.24]	0.0006 [0.06]	0.0006 [0.06]
VT1-VT2	0.0182	0.0504	2.2	5.6	6.1	0.999 (0.999–0.999)	0.998 [0.24]	0.0006 [0.06]	0.0004 [0.04]
**>1.20 m·s^−1^**									
TF-VT1	0.0447	0.1240	3.2	6.4	6.8	0.963 (0.933–0.980)	0.931 [6.89]	0.0031 [0.31]	0.0006 [0.06]
TF-VT2	0.0548	0.1518	3.9	7.8	7.6	0.918 (0.851–0.955)	0.857 [14.31]	0.0063 [0.63]	0.0007 [0.07]
VT1-VT2	0.0280	0.0777	2.0	9.4	9.0	0.980 (0.964–0.989)	0.960 [4.03]	0.0016 [0.16]	0.0010 [0.10]
**1.20–0.95 m·s^−1^**									
TF-VT1	0.0316	0.0877	2.9	6.4	6.3	0.873 (0.753–0.935)	0.793 [20.74]	0.0024 [0.24]	0.0005 [0.05]
TF-VT2	0.0447	0.1240	4.1	6.2	6.3	0.790 (0.590–0.892)	0.698 [30.15]	0.0046 [0.46]	0.0005 [0.05]
VT1-VT2	0.0218	0.0605	2.0	6.4	6.3	0.950 (0.902–0.975)	0.906 [9.37]	0.0010 [0.10]	0.0005 [0.05]
**0.95–0.70 m·s^−1^**									
TF-VT1	0.0316	0.0877	3.8	7.6	7.5	0.930 (0.884–0.957)	0.978 [2.25]	0.0013 [0.13]	0.0007 [0.07]
TF-VT2	0.0336	0.0933	4.1	7.3	7.2	0.883 (0.807–0.929)	0.883 [11.72]	0.0023 [0.23]	0.0007 [0.07]
VT1-VT2	0.0133	0.0370	1.6	4.2	4.1	0.977 (0.961–0.986)	0.955 [4.49]	0.0004 [0.04]	0.0002 [0.02]
**0.70–0.45 m·s^−1^**									
TF-VT1	0.0206	0.0570	3.7	7.3	7.1	0.963 (0.940–0.977)	0.929 [7.12]	0.0009 [0.09]	0.0007 [0.07]
TF-VT2	0.0316	0.0877	5.4	7.0	6.9	0.948 (0.911–0.970)	0.903 [9.68]	0.0012 [0.12]	0.0006 [0.06]
VT1-VT2	0.0154	0.0426	2.7	5.3	5.3	0.973 (0.953–0.985)	0.947 [5.26]	0.0005 [0.05]	0.0004 [0.04]
**<0.45 m·s^−1^**									
TF-VT1	0.0097	0.03	3.0	2.9	3.1	0.992 (0.987–0.995)	0.984 [1.58]	0.0002 [0.02]	0.0001 [0.01]
TF-VT2	0.0128	0.04	4.0	3.1	3.4	0.986 (0.976–0.992)	0.973 [2.69]	0.0003 [0.03]	0.0001 [0.01]
VT1-VT2	0.0072	0.02	2.2	2.2	2.4	0.995 (0.992–0.997)	0.991 [0.91]	0.0001 [0.01]	0.0001 [0.01]

TF: T-Force; VT1: Vitruve device 1; VT2: Vitruve device 2; SEM: standard error of measurement; SDC: smallest detectable change (sensitivity); CV: SEM expressed as a coefficient of variation; SEE: standard error of the estimate; ME_SEE_: maximum error calculated from the SEE; ME_BIAS_: maximum error calculated from the Bland–Altman bias; ICC: intraclass correlation coefficient, model (1, k); CI: confidence interval; CCC: Lin’s concordance correlation coefficient; MSD: mean square deviation; VMD: variance of the difference between measurements; Dev: percent deviation from 1 (for CCC) or 0 (for MSD and VMD).

**Table 2:** Between-device agreement (reproducibility) for the maximal velocity in the bench press exercise according to the velocity range addressed.

	MAGNITUDE OF ERROR					AGREEMENT			
	SEM (m·s^−1^)	SDC (m·s^−1^)	CV (%)	ME_SEE_ (%1RM)	ME_BIAS_ (%1RM)	ICC (CI 95%)	CCC [DEV (%)]	MSD [DEV (%)]	VMD [DEV (%)]
**POOLED**									
TF-VT1	0.0792	0.2196	5.9	11.4	23.4	0.990 (0.988–0.993)	0.981 [1.89]	0.0129 [1.29]	0.0070 [0.70]
TF-VT2	0.0632	0.1753	4.6	11.8	20.5	0.995 (0.993–0.996)	0.990 [1.10]	0.0076 [0.76]	0.0053 [0.54]
VT1-VT2	0.0316	0.0877	2.4	8.8	8.3	0.998 (0.998–0.999)	0.997 [0.30]	0.0018 [0.18]	0.0009 [0.09]
**>1.20 m·s^−1^**									
TF-VT1	0.1643	0.4555	7.2	12.1	21.8	0.754 (0.551–0.865)	0.663 [33.67]	0.0531 [5.31]	0.0061 [0.61]
TF-VT2	0.1304	0.3614	5.6	12.4	20.1	0.847 (0.718–0.917)	0.760 [23.97]	0.0331 [3.31]	0.0051 [0.51]
VT1-VT2	0.0471	0.1306	2.1	14.7	8.2	0.972 (0.949–0.985)	0.947 [5.34]	0.0045 [0.45]	0.0008 [0.08]
**1.20–0.95 m·s^−1^**									
TF-VT1	0.0775	0.2147	4.4	7.2	9.9	0.832 (0.672–0.914)	0.663 [33.67]	0.0130 [1.30]	0.0061 [0.61]
TF-VT2	0.0571	0.1581	3.2	9.1	10.2	0.919 (0.840–0.959)	0.760 [23.97]	0.0065 [0.65]	0.0051 [0.51]
VT1-VT2	0.0314	0.0869	1.8	8.8	7.6	0.970 (0.941–0.985)	0.947 [5.34]	0.0020 [0.20]	0.0008 [0.08]
**0.95–0.70 m·s^−1^**									
TF-VT1	0.0483	0.1339	3.6	9.8	10.1	0.941 (0.904–0.964)	0.893 [10.73]	0.0047 [0.47]	0.0013 [0.13]
TF-VT2	0.0316	0.0877	2.3	8.8	9.6	0.977 (0.963–0.986)	0.956 [4.44]	0.0018 [0.18]	0.0013 [0.13]
VT1-VT2	0.0289	0.0802	2.2	7.5	6.5	0.976 (0.961–0.985)	0.954 [4.59]	0.0017 [0.17]	0.0005 [0.05]
**0.70–0.45 m·s^−1^**									
TF-VT1	0.0289	0.0800	2.9	9.8	7.7	0.978 (0.962–0.987)	0.964 [0.95]	0.0014 [0.14]	0.0006 [0.02]
TF-VT2	0.0316	0.0877	3.1	10.1	6.2	0.986 (0.976–0.992)	0.987 [1.34]	0.0005 [0.05]	0.0005 [0.05]
VT1-VT2	0.0202	0.0561	2.0	6.2	5.4	0.988 (0.980–0.993)	0.977 [2.28]	0.0008 [0.08]	0.0004 [0.04]
**<0.45 m·s^−1^**									
TF-VT1	0.0127	0.0351	1.9	4.2	6.2	0.995 (0.992–0.997)	0.991 [0.95]	0.0003 [0.03]	0.0002 [0.02]
TF-VT2	0.0124	0.0343	1.9	5.2	4.6	0.996 (0.993–0.998)	0.991 [1.34]	0.0003 [0.03]	0.0003 [0.05]
VT1-VT2	0.0175	0.0485	2.6	6.1	5.5	0.991 (0.984–0.995)	0.981 [1.86]	0.0006 [0.06]	0.0004 [0.04]

TF: T-Force; VT1: Vitruve device 1; VT2: Vitruve device 2; SEM: standard error of measurement; SDC: smallest detectable change (sensitivity); CV: SEM expressed as a coefficient of variation; SEE: standard error of the estimate; MESEE: maximum error calculated from the SEE; MEBIAS: maximum error calculated from the Bland–Altman bias; ICC: intraclass correlation coefficient, model (1, k); CI: confidence interval; CCC: Lin’s concordance correlation coefficient; MSD: mean square deviation; VMD: variance of the difference between measurements; Dev: percent deviation from 1 (for CCC) or 0 (for MSD and VMD).

**Table 3:** Between-device agreement (reproducibility) for the mean propulsive velocity in the full squat exercise according to the velocity range assessed.

	MAGNITUDE OF ERROR					AGREEMENT			
	SEM (m·s^−1^)	SDC (m·s^−1^)	CV (%)	ME_SEE_ (%1RM)	ME_BIAS_ (%1RM)	ICC (CI 95%)	CCC [DEV (%)]	MSD [DEV (%)]	VMD [DEV (%)]
**POOLED**									
TF-VT1	0.0226	0.0626	2.0	7.5	7.5	0.998 (0.997–0.998)	0.996 [0.41]	0.0010 [0.10]	0.0007 [0.07]
TF-VT2	0.0169	0.0469	1.5	6.6	6.6	0.999 (0.999–0.999)	0.998 [0.24]	0.0006 [0.06]	0.0006 [0.06]
VT1-VT2	0.0173	0.0479	1.6	5.4	5.5	0.999 (0.999–0.999)	0.998 [0.24]	0.0006 [0.06]	0.0004 [0.04]
**>1.50 m·s^−1^**									
TF-VT1	0.0314	0.0871	1.9	11.4	13.0	0.946 (0.912–0.967)	0.897 [10.28]	0.0015 [0.15]	0.0016 [0.16]
TF-VT2	0.0311	0.0863	1.9	12.8	14.5	0.926 (0.874–0.956)	0.859 [14.05]	0.0020 [0.20]	0.0020 [0.20]
VT1-VT2	0.0353	0.0977	2.2	13.4	15.8	0.916 (0.856–0.951)	0.844 [15.62]	0.0025 [0.25]	0.0024 [0.24]
**1.50–1.25 m·s^−1^**									
TF-VT1	0.0316	0.0877	2.3	11.8	10.6	0.909 (0.860–0.941)	0.836 [16.39]	0.0023 [0.23]	0.0014 [0.14]
TF-VT2	0.0269	0.0744	2.0	10.6	10.6	0.940 (0.906–0.962)	0.885 [11.46]	0.0014 [0.15]	0.0014 [0.14]
VT1-VT2	0.0210	0.0581	1.5	5.9	6.1	0.961 (0.939–0.975)	0.926 [7.37]	0.0004 [0.04]	0.0005 [0.05]
**1.25–1.00 m·s^−1^**									
TF-VT1	0.0192	0.0531	1.7	5.9	6.0	0.964 (0.945–0.976)	0.936 [6.43]	0.0007 [0.07]	0.0005 [0.05]
TF-VT2	0.0140	0.0389	1.3	5.6	5.6	0.981 (0.972–0.988)	0.963 [3.68]	0.0004 [0.04]	0.0004 [0.04]
VT1-VT2	0.0184	0.0510	1.7	5.6	5.5	0.968 (0.952–0.979)	0.978 [2.22]	0.0007 [0.07]	0.0004 [0.04]
**1.00–0.75 m·s^−1^**									
TF-VT1	0.0192	0.0531	2.2	5.3	5.3	0.966 (0.949–0.978)	0.936 [6.43]	0.0007 [0.07]	0.0004 [0.04]
TF-VT2	0.0162	0.0451	1.8	6.2	6.2	0.974 (0.961–0.983)	0.950 [5.02]	0.0005 [0.05]	0.0005 [0.05]
VT1-VT2	0.0144	0.0398	1.6	4.5	4.6	0.980 (0.970–0.987)	0.962 [3.83]	0.0001 [0.01]	0.0003 [0.03]
**<0.75 m·s^−1^**									
TF-VT1	0.0192	0.0531	3.2	2.8	2.9	0.992 (0.988–0.995)	0.985 [1.54]	0.0002 [0.02]	0.0001 [0.01]
TF-VT2	0.0076	0.0211	1.3	2.8	2.8	0.996 (0.994–0.998)	0.992 [0.77]	0.0001 [0.01]	0.0001 [0.01]
VT1-VT2	0.0098	0.0270	1.6	3.1	3.2	0.996 (0.993–0.997)	0.991 [0.87]	0.0002 [0.02]	0.0001 [0.01]

TF: T-Force; VT1: Vitruve device 1; VT2: Vitruve device 2; SEM: standard error of measurement; SDC: smallest detectable change (sensitivity); CV: SEM expressed as a coefficient of variation; SEE: standard error of the estimate; MESEE: maximum error calculated from the SEE; MEBIAS: maximum error calculated from the Bland–Altman bias; ICC: intraclass correlation coefficient, model (1, k); CI: confidence interval; CCC: Lin’s concordance correlation coefficient; MSD: mean square deviation; VMD: variance of the difference between measurements; Dev: percent deviation from 1 (for CCC) or 0 (for MSD and VMD).

**Table 4:** Between-device agreement (reproducibility for the maximal velocity in the full squat exercise according to the velocity range assessed.

	MAGNITUDE OF ERROR					AGREEMENT			
	SEM (m·s^−1^)	SDC (m·s^−1^)	CV (%)	ME_SEE_ (%1RM)	ME_BIAS_ (%1RM)	ICC (CI 95%)	CCC [DEV (%)]	MSD [DEV (%)]	VMD [DEV (%)]
**POOLED**									
TF-VT1	0.0894	0.2479	5.0	12.1	18.9	0.966 (0.958–0.972)	0.937 [6.34]	0.0165 [1.65]	0.0046 [0.46]
TF-VT2	0.0847	0.2347	4.1	26.1	22.4	0.970 (0.963–0.975)	0.953 [4.56]	0.0114 [1.14]	0.0064 [0.64]
VT1-VT2	0.0548	0.1518	3.1	23.2	17.9	0.986 (0.982–0.988)	0.976 [2.52]	0.0054 [0.54]	0.0041 [0.41]
**>1.50 m·s^−1^**									
TF-VT1	0.1414	0.3925	6.2	6.5	20.7	0.695 (0.516–0.809)	0.611 [38.88]	0.0409 [4.09]	0.0054 [0.54]
TF-VT2	0.1517	0.4204	6.6	6.1	38.8	0.696 (0.506–0.813)	0.588 [41.20]	0.0466 [4.66]	0.0192 [1.92]
VT1-VT2	0.0915	0.2535	4.2	7.8	36.1	0.849 (0.756–0.907)	0.736 [26.42]	0.0170 [1.70]	0.0166 [1.66]
**1.50–1.25 m·s^−1^**									
TF-VT1	0.1000	0.2772	5.0	13.1	20.7	0.812 (0.710–0.878)	0.611 [38.88]	0.0409 [4.09]	0.0054 [0.54]
TF-VT2	0.0861	0.2386	4.3	41.2	38.8	0.866 (0.790–0.914)	0.588 [41.20]	0.0475 [4.66]	0.0192 [1.92]
VT1-VT2	0.0716	0.1984	3.7	42.5	36.1	0.882 (0.816–0.925)	0.736 [26.42]	0.0170 [1.70]	0.0166 [1.66]
**1.25–1.00 m·s^−1^**									
TF-VT1	0.0775	0.2150	4.4	13.1	11.8	0.885 (0.827–0.923)	0.810 [19.03]	0.0123 [1.23]	0.0018 [0.18]
TF-VT2	0.0708	0.1962	4.0	29.1	22.1	0.923 (0.884–0.949)	0.860 [14.03]	0.0101 [1.01]	0.0062 [0.62]
VT1-VT2	0.0316	0.0877	1.8	30.1	8.5	0.986 (0.978–0.990)	0.972 [2.84]	0.0020 [0.20]	0.0009 [0.09]
**1.00–0.75 m·s^−1^**									
TF-VT1	0.0624	0.17	4.0	9.1	12.4	0.903 (0.851–0.937)	0.834 [16.65]	0.0078 [0.78]	0.0020 [0.20]
TF-VT2	0.0447	0.1240	2.9	25.8	10.3	0.958 (0.935–0.973)	0.921 [7.92]	0.0035 [0.35]	0.0013 [0.13]
VT1-VT2	0.0316	0.0877	2.1	9.8	7.1	0.977 (0.964–0.985)	0.955 [4.50]	0.0016 [0.16]	0.0006 [0.06]
**<0.75 m·s^−1^**									
TF-VT1	0.0438	0.1213	3.2	11.1	8.2	0.920 (0.871–0.950)	0.859 [14.06]	0.0038 [0.38]	0.0009 [0.09]
TF-VT2	0.0316	0.0877	2.3	9.1	7.1	0.970 (0.951–0.981)	0.942 [5.80]	0.0015 [0.15]	0.0006 [0.06]
VT1-VT2	0.0316	0.0877	2.3	8.5	6.8	0.971 (0.954–0.983)	0.945 [5.51]	0.0012 [0.12]	0.0006 [0.06]

TF: T-Force; VT1: Vitruve device 1; VT2: Vitruve device 2; SEM: standard error of measurement; SDC: smallest detectable change (sensitivity); CV: SEM expressed as a coefficient of variation; SEE: standard error of the estimate; MESEE: maximum error calculated from the SEE; MEBIAS: maximum error calculated from the Bland–Altman bias; ICC: intraclass correlation coefficient, model (1, k); CI: confidence interval; CCC: Lin’s concordance correlation coefficient; MSD: mean square deviation; VMD: variance of the difference between measurements; Dev: percent deviation from 1 (for CCC) or 0 (for MSD and VMD).

## Data Availability

The data presented in this study are available on request from the corresponding author. The data is not publicly available due to confidentiality.
